# Live Oral Adenovirus Type 4 and Type 7 Vaccine Induces Durable Antibody Response

**DOI:** 10.3390/vaccines8030411

**Published:** 2020-07-23

**Authors:** Natalie D. Collins, Anima Adhikari, Yu Yang, Robert A. Kuschner, Nicos Karasavvas, Leonard N. Binn, Shannon D. Walls, Paul C. F. Graf, Christopher A. Myers, Richard G. Jarman, Jun Hang

**Affiliations:** 1 Viral Diseases Branch, Walter Reed Army Institute for Research, Silver Spring, MD 20910, USA; anima.adhikari.ctr@mail.mil (A.A.); yu.yang2.ctr@mail.mil (Y.Y.); robert.a.kuschner.ctr@mail.mil (R.A.K.); Nkarasavva@hivresearch.org (N.K.); leonard.n.binn.vol@mail.mil (L.N.B.); shannon.d.walls6.mil@mail.mil (S.D.W.); richard.g.jarman.mil@mail.mil (R.G.J.); jun.hang.civ@mail.mil (J.H.); 2 Naval Health Research Center, San Diego, CA 92186, USA; paul.c.graf2.mil@mail.mil (P.C.F.G.); christopher.a.myers48.civ@mail.mil (C.A.M.); 3 U.S. Navy Medical Research Unit Six, Lima 07006, Peru

**Keywords:** human adenovirus, live vaccine, human respiratory illness, acute respiratory disease, immunity, serology

## Abstract

Human adenoviruses (AdV) are mostly associated with minimal pathology. However, more severe respiratory tract infections and acute respiratory diseases, most often caused by AdV-4 and AdV-7, have been reported. The only licensed vaccine in the United States, live oral AdV-4 and AdV-7 vaccine, is indicated for use in the military, nearly exclusively in recruit populations. The excellent safety profile and prominent antibody response of the vaccine is well established by placebo-controlled clinical trials, while, long-term immunity of vaccination has not been studied. Serum samples collected over 6 years from subjects co-administered live oral AdV-4 and AdV-7 vaccine in 2011 were evaluated to determine the duration of the antibody response. Group geometric mean titers (GMT) at 6 years post vaccination compared to previous years evaluated were not significantly different for either AdV-4 or AdV-7 vaccine components. There were no subjects that demonstrated waning neutralization antibody (NAb) titers against AdV-4 and less than 5% of subjects against AdV-7. Interestingly, there were subjects that had a four-fold increase in NAb titers against either AdV-4 or AdV-7, at various time points post vaccination, suggesting either homotypic or heterotypic re-exposure. This investigation provided strong evidence that the live oral AdV-4 and AdV-7 vaccine induced long-term immunity to protect from AdV-4 and AdV-7 infections.

## 1. Introduction

Human adenovirus (AdV) infections by a variety of different types are common, particularly during childhood. AdVs were originally defined serologically and currently by genomic relatedness [1,2,3]. AdV related illnesses in healthy individuals are usually mild and self-limited, but severe infections of the lower respiratory tract occasionally occur and can be fatal [1,4]. In the U.S. military, large outbreaks of acute respiratory disease (ARD) frequently occurred in new recruits during their initial training and were typically caused by types 4 and 7. Development and deployment of the AdV-4 and AdV-7 vaccine was paramount in controlling AdV-4- and AdV-7-related ARD in recruits. The vaccine is administered orally to all new recruits and causes an asymptomatic infection of the gastrointestinal tract [5,6,7,8]. Vaccination or natural infection induces production of serum neutralizing antibodies (NAb) which are associated with protection from re-infection with the same type but, in general, do not provide cross-protection against heterologous types [9]. The duration of protection following natural infection or vaccination is believed to be long-lasting, largely based upon the absence of reported outbreaks in previously vaccinated military populations and the observation that natural infections only occur in individuals with no or very low levels of serum neutralizing antibodies.

Serum antibodies have been identified up to eight weeks after vaccination, which is the approximate duration of basic training and the period of highest risk for members of the military [7,8]. However, data on long-term vaccine-induced antibody response is limited. AdV-4 and AdV-7 respiratory infections are rarely reported outside of basic training settings but it is unclear if this is due to long-term protection of the vaccine or simply due to absence of exposure to these AdV types after basic training [10]. If the vaccine affords durable protection, large ARD outbreaks are unlikely to occur and re-vaccination of service members should not be necessary. In this study, pre- and post-vaccination serum samples collected over 6 years following AdV vaccination were assayed to determine the duration of the neutralizing antibody response to AdV-4 and AdV-7 to inform vaccination strategies.

## 2. Materials and Methods

### 2.1. Study Population

Paired serum from 60 subjects collected prior to vaccination (pre-vaccination) and three post vaccination samples up to 6 years (>30 days to one year, two to three years, and five to 6 years) after co-administering live oral AdV-4 and AdV-7 vaccine in 2011 were obtained from the Department of Defense Serum Repository (DoDSR), maintained by the Armed Forces Health Surveillance Branch (AFHSB), Department Health Agency (DHA) [11,12], for a total of 240 samples (Table S1). DoDSR serum samples are collected for operational and epidemiologic purposes as part of the Defense Medical Surveillance System (DMSS), a command-directive, public health surveillance practice; samples are accessible only to Department of Defense investigators [11,12].

### 2.2. Colorimetric Microneutralization Assay

Pre-immunization, and three post vaccination serum samples for each subject were evaluated utilizing a modification of the colorimetric neutralization assay described previously to determine neutralizing antibody titers against AdV-4 strain RI-67 and AdV-7 strain 7a [13]. Subject sera, subtype specific positive reference sera (human and rabbit) and rabbit negative reference sera were heat inactivated at 56 °C ± 1 °C for 30 min. Heat-inactivated sera were either serially diluted two-fold in triplicate for subject sera from 1:4 to 1:256 (subjects whose 50% NAb titer (NT50) exceeded 256 were diluted 1:4 to 1:8195), human reference sera and positive rabbit reference sera or ten-fold for negative rabbit sera in 1× PBS and incubated with 200 TCID_50_ of either AdV-4 strain RI-67 or AdV-7 strain 7a in 96-well tissue culture plates to generate virus-antibody complexes at 37 °C in 5% CO_2_ for 1 h. A549 cells, at a density of 2.0 × 10^4^ cells per well, were added to virus-antibody complexes and plates incubated for an additional 7 days at 37 °C in 5% CO_2_, then a 1:5 neutral red solution, diluted in 1 × PBS, was added to the wells and plates incubated for 60–75 min at 37 °C in 5% CO_2_. Plates were then washed three times with 1 × PBS, lysed with 50% isopropyl alcohol, 49% tissue culture grade water and 1% glacial acetic acid, and read at 550 nm to measure colorimetric cytopathic effect of cells. The NT50 for subjects was determined by nonlinear regression in GraphPad Prism version 8.1.1 (GraphPad Software, San Diego, CA, USA) and log_10_ transformed. NT50 greater than or equal to 4 indicated presence of AdV-specific neutralizing antibodies.

### 2.3. Statistical Analysis

Data analysis was conducted in GraphPad Prism version 8.1.1. Group geometric mean titer (GMT) of pre-immunization and each timeframes post-immunization were determined utilizing the NT50 calculated by nonlinear regression; subjects without a detectable NAb titer (NT50 < 4) are represented as NT50 2. GMTs were then compared by an ANOVA with Tukey’s multiple comparisons test when appropriate and *p* < 0.05 required for significance.

## 3. Results

### 3.1. Significant Antibody Response in the Study Population After Vaccination

The study population of military recruits given the AdV vaccine as part of the normal vaccination program consisted of 32 males and 28 females aged 17 to 34 at initial vaccination. Serostatus against AdV-4 and AdV-7 prior to vaccination was determined for all 60 subjects (Table 1 and Table S1). Eighteen of 60 subjects (30%) were seronegative prior to vaccination for either AdV-4 or AdV-7; majority seroconverted, 18 of 18 against AdV-4 and 16 of 18 against AdV-7, with GMTs of 26 against AdV-4 and 69 against AdV-7 after vaccination. Forty-two of 60 subjects (70%) were seropositive prior to vaccination for either AdV-4 or AdV-7. For subjects that were seropositive prior to vaccination, greater than 40% demonstrated an increase by four-fold or greater in their NT50 and GMTs were significantly increased from 8 to 110 against AdV-4 and 16 to 120 against AdV-7. Collectively, regardless of pre-vaccination serostatus, subjects demonstrated a significant antibody response following AdV-4 and AdV-7 vaccination.

### 3.2. Persistence of Neutralizing Antibody Response to Immunization

**Figure 1:**
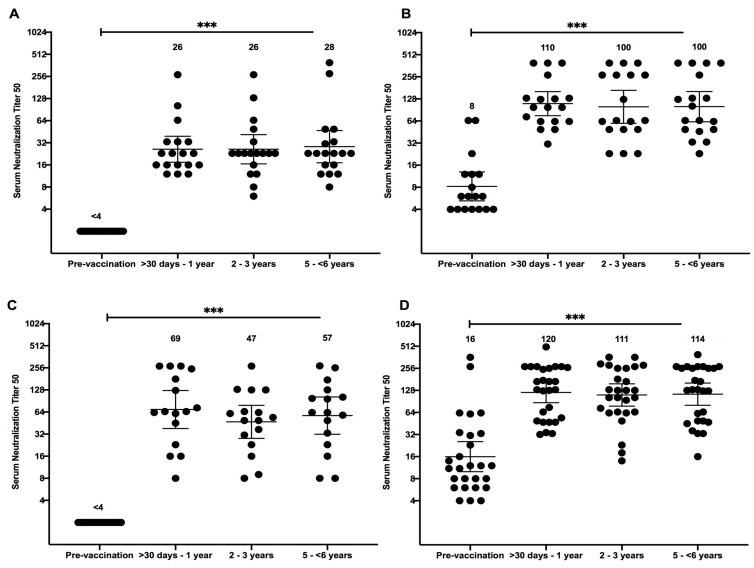
Individual neutralizing antibody titer against Adenovirus type 4 and type 7 with geometric means at 95% confidence interval according to time. Neutralizing antibody (NAb) titers of subjects given single dose of live oral adenovirus (AdV)-4 and AdV-7 vaccine by years, post-vaccination (N = 36 for AdV-4 and 41 for AdV-7), 50% NAb titer (NT50) was determined by colorimetric cytopathic effect based neutralization assay against AdV-4 for seronegative (**A**) and seropositive subjects (**B**) and against AdV-7 for seronegative (**C**) and seropositive (**D**) subjects. NT50 equal to or greater than 4 demonstrates seroconversion and a positive. Statistical significance is indicated as follows: ns, not significant (*p* value = 0.12), *, *p* = 0.033; **, *p* = 0.002; ***, *p* < 0.001.

Subjects that seroconverted or had a four-fold increase from baseline NAb titer to either AdV-4 or AdV-7 one year post-vaccination were further evaluated to determine vaccine durability (N = 36 for AdV-4 and 41 for AdV-7). Four-fold decrease, which would demonstrate waning immunity, was observed in less than 5% of subjects and only against AdV-7 (Table 2 and Table S1). At two to three years post-vaccination the NT50 for subject ADV032 waned from 270 to 63. At five to 6 years post-vaccination the NT50 for subject ADV002 waned from 282 to 33, while subject ADV046 waned from 270 to 36. However, all of these subjects still had NT50 greater than 4, presumably having sufficient protection against AdV-7 infection. GMTs were calculated by pre-vaccination serostatus, separately, for both AdV-4 and AdV-7. The type specific GMTs against AdV-4 for both seronegative and seropositive subjects did not differ significantly when compared to each other; the GMT was 26 for seronegative subjects and 110 for seropositive subjects at >30 days–1 year, 26 for seronegative subjects and 100 for seropositive subjects at two to three years, and 28 for seronegative subjects and 100 for seropositive subjects at five to 6 years (Figure 1A,B). Similarly, GMTs against AdV-7 remained consistent post-vaccination (Figure 1); the GMT was 69 for seronegative subjects and 120 for seropositive subjects at > 30 days–1 year, 47 for seronegative subjects and 111 for seropositive subjects at two to three years, and 57 for seronegative subjects and 114 for seropositive subjects at five to 6 years (Figure 1C,D). The results show that AdV-4- and AdV-7-specific antibodies persist for at least 6 years following vaccination.

Interestingly, a small proportion of subjects demonstrated a boost in immune response, evident by a four-fold increase in NT50, in years following vaccination (Table S1). Two subjects demonstrated an antibody boost against AdV-4; the NT50 for subject ADV0027 increased from 63 to 393 at two to three years post-vaccination, while the NT50 for subject ADV036 increased from 33 to 279 at five to 6 years post-vaccination. Three subjects demonstrated an antibody boost against AdV-7 at two to three years post vaccination; the NT50 for subject ADV029 increased from 65 to 294, subject ADV030 increased from 54 to 282, and subject ADV046 increased from 32 to 270. Subject ADV020 was the only subject that demonstrated a boost against AdV-7 at five to 6 years post vaccination; the NT50 increased from 18 to 126. The results suggest these subjects might have been re-exposed to an AdV in the years following the initial vaccination, though no medical records are available to support this observation.

## 4. Discussion

AdV-4 and AdV-7 associated ARD outbreaks were common in recruits when the vaccine were not available, but notably do not recur in service members after recruit training, even years later. This may be the result of long-lasting protection from the vaccine or due to limited exposure to the viruses outside of the recruit setting. Isolated cases and small clusters of illness outside military recruit setting have been identified. It is not known if they were either due to lack of vaccination and no naturally occurring protective serum antibodies or waning vaccine immunity. This is the first study to assess the long-term duration of vaccine-induced serum neutralizing antibodies, which are highly correlated with protection from clinical disease due to AdV-4 and AdV-7. Our analysis shows all subjects who either seroconverted in response to the vaccine or whose antibody response was boosted following vaccination still had detectable antibodies at 6 years post-vaccination. Further, evidence of waning antibodies after vaccination was observed in only three study subjects and all maintained detectable antibodies. This study provides for the first time, evidence that vaccine-specific antibodies persist for at least 6 years, bolstering that the AdV vaccine induces long-term immunity.

A limitation of this study is the lack of clinical data. Investigators were purposefully blinded to clinical data of the subjects as a condition of sample usage with AFHSB, DHA [12]. An additional limitation is the number of subjects evaluated. All subjects who fulfilled the criteria for the study and available at DoDSR were selected, which were vaccinated with live oral AdV-4 and AdV-7 after re-introduction of the vaccine in 2011 with consecutive serum samples at the specified time frames of pre-vaccination, >30 days to one year, two to three years, and five to 6 years.

The serostatus for all 60 subjects in this study was determined and approximately 70% of study subjects were seropositive to either AdV-4 or AdV-7 prior to vaccination. The observed serostatus for AdV-7 is consistent with earlier reports, while the serostatus for AdV-4 was approximately 35% higher than in past reports [7,8,14]. The serostatus of military recruits varies across studies and the majority of published seroprevalence studies were conducted over 10 years ago [7,8,14,15]. Differences in serum neutralization assay procedures contribute to variability in seroprevalence in military recruits. Our sample size was smaller than previous published studies because we selected individuals with a pre-vaccination sample as well as at least three consecutive post-vaccination samples collected over 6 years of active duty service. Our study population was selected randomly from a large military serum repository, therefore, a likely representative of the larger military population.

Persistent antibodies, for at least 6 years, were detected in 36 and 41 subjects against AdV-4 and AdV-7, respectively, who either seroconverted or had a four-fold increase in NAb titers. Three subjects against AdV-7 (ADV002, ADV032, ADV046) showed evidence of waning antibody titers, demonstrated by a four-fold decrease in NAb titer. The level of antibodies necessary for protection has not been definitively established for either AdV-4 or AdV-7. Earlier studies suggested that any detectable antibody titer following vaccination provided some protection from ARD and NAb titer levels higher than 16 are typically completely protective [16,17,18]. The NAb response for only one of the three subjects who warned against AdV-7 dropped below 36. Therefore, it is likely that all subjects that waned against AdV-7 would still be protected. Additional studies to determine the NAb titer for protection from AdV-7 associated ARD are needed to support this claim.

Because of universal vaccination of military recruits, ARD surveillance studies demonstrate AdV- 4 and AdV-7 disease occurs only rarely in this population. Studies have shown that heterotypic responses to infection from related AdVs may boost antibody titers [17,19]. Further, cross-neutralization studies conducted with reference antisera supports that sera generated against one AdV serotype cross-reacts with other AdV serotypes, demonstrating that heterotypic infections may contribute to observed antibody responses [9,20,21,22]. AdV-4 is the only human species E AdV, however, AdV-16 reference antisera has been shown to cross-neutralize AdV-4; attributed to inclusion of simian AdV-16 genome in the hexon region of AdV-4 [9,20,21,22,23]. Species B consists of ten human AdV of medical importance and AdV-11, AdV-14, AdV-21, and AdV-55 have all been associated with significant episodic disease in military populations [24,25,26,27,28,29]. Because of the lack of clinical data on the subjects, which was mandated by the AFHSB, we are unable to determine if an AdV infection subsequent to their vaccination was documented in their medical records. Further, we are unaware of AdV-4 and AdV-7 outbreaks in military populations over the time course of the study period. Therefore, distinguishing subsequent either homotypic or heterotypic infection that may have contributed to antibody boost observed for the six subjects (ADV020, ADV027, ADV029, ADV30, ADV036, and ADV046) would be difficult.

The live oral AdV-4 and AdV-7 vaccine are well-established as safe and efficacious [30,31], and our study demonstrates antibody protection is durable. These attributes are likely due to the fact that the vaccine viruses are unattenuated and administrated orally to replicate in the GI tract. Immunoglobulin (Ig) M, G, and A sub-classes have been studied following natural respiratory infection [32,33]. Studies on Ig sub-classes induced following live oral AdV-4 and type AdV-7 vaccination and protection from ARD are limited. However, earlier studies on secretary Ig following either live oral AdV-21 or live oral AdV-4 vaccination found no evidence of IgA in nasal passages of vaccine recipients, suggesting that IgA may explain protection from ARD, even though IgA was detected in both sera and feces of vaccine recipients [34,35]. Similarly, IgG and IgM were also not detected in nasal passages of vaccine recipients [34,35]. Detectable neutralizing antibody titers following live oral AdV-4 and AdV-7 correlates with protection, the mechanism is unclear and additional studies are warranted. Oral vaccination with AdV-4 and AdV-7 is feasible because these “respiratory” viruses infect the GI tract without causing illness. However, vaccine recipients shed infectious virus in the stool as long as 28 days following vaccination [7]. This is not a safety issue in recruits because they are all healthy, non-pregnant, and receive the vaccine together at the beginning of their training. Civilian groups are also susceptible to occasional outbreaks of AdV-associated ARD [36,37]. However, any use in civilian populations poses additional risks because of the possibility of fecal transmission to individuals who are immunosuppressed, pregnant, very young or elderly [7,8]. Therefore, transmission of vaccine virus due to fecal shedding needs to be considered in settings where close contacts cannot be controlled.

## 5. Conclusions

This study demonstrates AdV-4 and AdV-7 vaccination produces a robust and durable immunological response in military recruits and provides sufficient protection from AdV-4- and AdV-7-related respiratory disease for at least 6 years. The long-term protection afforded by the live oral AdV-4 and AdV-7 vaccine likely contributes to minimal ARD related to these pathogens in congregated military settings where both vaccinated and unvaccinated populations are present. Continued serological studies to further evaluate vaccine durability beyond 6 years post-vaccination is relevant as military careers may continue for as long as twenty or more years.

## Figures and Tables

**Table 1:** Baseline serostatus and seroconversion following live oral adenovirus-4 and 7 vaccination 30 days to 1 year.

Vaccination and Immune Status	Adenovirus-4		Adenovirus-7	
	Seronegative (% or CI)	Seropositive (% or CI)	Seronegative (% or CI)	Seropositive (% or CI)
Baseline serostatus	18 (30%)	42 (70%)	18 (30%)	42 (70%)
Seroconversion/4-fold increase *	18 (100%)	18 (43%)	16 (89%)	25 (60%)
Pre-vaccination GMT	<4	8 (5–13)	<4	16 (10–26)
<30–1 year GMT	26 (17–39)	110 (76–161)	69 (38–126)	120 (87–165)

* Determined as a detectable 50% neutralizing antibody titers (NT50) ≥ 4 for seronegative subjects or a 4-fold increased from baseline NT50 compared to NT50 at <30 days–1 year for seropositive subjects. GMT = geometric mean titer and only determine for subjects with vaccine specific antibody response. CI = lower and upper 95% confidence interval for GMT.

**Table 2:** Serostatus for subjects that demonstrated a vaccine specific antibody response following vaccination by years post vaccination.

Time Post-Vaccination	Adenovirus-4			Adenovirus-7		
	Seropositive *	Subjects with 4-Fold decrease	Subjects with 4-Fold increase	Seropositive *	Subjects with 4-Fold decrease	Subjects with 4-Fold increase
>30 Days–1 Year	36 (100%)	0 (0%)	0 (0%)	41(100%)	0 (0%)	0 (0%)
2–3 Years	36 (100%)	0 (0%)	1 (2.8%)	41 (100%)	1 (2.4%)	3 (7.3%)
5–6 Years **	36 (100%)	0 (0%)	1 (2.8%)	41 (100%)	2 (4.9%)	1 (2.4%)

* Neutralizing antibody titers ≥4 are considered positive using the colorimetric neutralization assay with a 50% cutoff. ** Maximum years post-vaccination was 6 years. GMT, geometric mean titer. CI, lower and upper 95% confidence interval for GMT.

## Supplementary Materials

The following are available online at https://www.mdpi.com/2076-393X/8/3/411/s1, Table S1: Neutralizing antibody titer for all subjects following live oral adenovirus-4 and adenovirus-7 vaccination.

## Disclaimer

Material has been reviewed by the authors’ respective institutions. There is no objection to its presentation and/or publication. The views expressed here are those of the authors and do not reflect the official policy of the U.S. Department of the Army, Department of the Navy, Department of Defense or U.S. Government. This is the work of U.S. government employees and may not be copyrighted (17 USC 105).
