# Supplementary material for: Live Oral Adenovirus Type 4 and Type 7 Vaccine Induces Durable Antibody Response
Source: Vaccines (Basel). 2020 Jul 23;8(3):411. doi: 10.3390/vaccines8030411 (PMC7564809; doi:10.3390/vaccines8030411)
Supplement: Supplementary file 1 [file vaccines-08-00411-s001.pdf]

Table 1. Neutralizing antibody titer for all subjects at baseline and following live oral adenovirus-4 and adenovirus-7 vaccination.

Subject	Adenovirus-4				Adenovirus-7			
	Pre-vaccination	>30 Days–1 Year	2–3 Years	5–6 Years	Pre-vaccination	>30 Days–1 Year	2–3 Years	5–6 Years
ADV001	6	23	23	63	12	169	102	49
ADV002	4	31	23	49	14	175	282	33 *
ADV003	6	23	23	47	<4	16	16	8
ADV004	16	31	63	65	<4	248	128	256
ADV005	4	63	49	126	6	248	363	270
ADV006	12	131	126	270	1005	1045	1167	741
ADV007	6	12	12	16	6	23	63	31
ADV008	4	128	63	46	73	49	362	502
ADV009	<4	270	270	393	<4	45	128	63
ADV010	128	393	1000	393	6	8	12	8
ADV011	65	270	270	393	63	270	102	270
ADV012	4	393	393	393	393	393	393	393
ADV013	<4	102	131	49	8	169	256	128
ADV014	6	49	23	23	<4	126	65	128
ADV015	393	393	393	270	61	262	73	131
ADV016	102	393	393	251	362	256	128	256
ADV017	12	131	270	102	<4	8	8	8
ADV018	6	126	65	65	<4	61	37	63
ADV019	6	98	49	49	270	128	182	256
ADV020	126	270	270	131	8	47	18	126 **
ADV021	126	128	126	270	16	15	23	34
ADV022	6	12	12	8	8	47	49	33
ADV023	65	393	393	393	175	128	270	128
ADV024	12	49	23	33	393	270	270	270
ADV025	102	393	393	393	12	49	94	270
ADV026	6	393	270	131	16	270	128	270
ADV027	4	63 **	393	131	<4	65	49	33
ADV028	270	393	393	393	47	63	73	91
ADV029	4	98	65	65	8	65	294 **	131
ADV030	8	73	270	393	4	54	282 **	270
ADV031	<4	23	23	23	23	131	102	175
ADV032	23	33	31	49	<4	270	63 *	102
ADV033	<4	23	23	33	502	393	393	502
ADV034	270	126	126	98	11	75	169	256
ADV035	131	126	126	270	<4	16	9	23
ADV036	<4	33	33	279 **	<4	65	61	96
ADV037	270	393	393	502	33	502	256	393
ADV038	<4	33	49	23	4	47	63	49
ADV039	<4	16	12	12	63	270	362	256
ADV040	270	270	126	131	47	126	175	270
ADV041	<4	12	8	8	270	393	393	270
ADV042	23	33	23	33	33	31	41	34
ADV043	23	23	33	65	34	270	128	65
ADV044	393	270	270	393	6	33	23	62
ADV045	49	128	98	126	11	131	65	47
ADV046	<4	16	23	31	6	32	270 **	36 *

ADV047	<4	16	23	23	<4	<4	4	<4
ADV048	49	98	49	49	16	26	34	31
ADV049	<4	12	23	23	6	169	63	16
ADV050	<4	23	23	23	<4	180	54	58
ADV051	<4	65	65	49	<4	63	31	16
ADV052	<4	12	6	12	<4	23	23	49
ADV053	<4	33	23	23	<4	270	130	175
ADV054	<4	16	12	12	<4	<4	<4	<4
ADV055	<4	16	23	16	12	270	131	126
ADV056	63	49	98	98	4	34	14	45
ADV057	4	63	49	33	270	131	128	62
ADV058	<4	23	16	16	<4	73	49	98
ADV059	23	98	63	63	<4	270	270	272
ADV060	49	65	65	63	31	126	65	169

* 4-fold decreased from previous time point that are within dilution range of 1:4 to 1:8195. ** 4-fold increase from previous time point that are within dilution range of 1:4 to 1:8195.
